# The incidence of medical attention injuries in wheelchair basketball at the London 2012 and Rio 2016 Paralympic Games: a descriptive sub-study

**DOI:** 10.17159/2078-516X/2025/v37i1a18449

**Published:** 2025-05-15

**Authors:** R Brombacher, M Eken, W Derman, P Runciman

**Affiliations:** 1 Division of Sport and Exercise Medicine, Department of Exercise, Sport and Lifestyle Medicine, Faculty of Medicine and Health Sciences, Stellenbosch University, South Africa; 2 International Olympic Committee Research Centre, Cape Town, South Africa

**Keywords:** Para-athlete, epidemiology, injury, Paralympics, surveillance

## Abstract

**Background:**

Wheelchair basketball is one of the most popular Para sports globally. However, there is limited literature on the epidemiology of injuries sustained by wheelchair basketball players.

**Objectives:**

This study aimed to describe injuries sustained by Paralympic athletes participating in wheelchair basketball at the London 2012 and Rio 2016 Paralympic Games.

**Methods:**

This study is a sub-study of the ongoing Paralympic injury and illness surveillance studies. Injury information from 430 athletes who participated in wheelchair basketball at the two Paralympic Games was analysed. Injuries were described by overall incidence, age, sex, onset, anatomical area, type of impairment, and time loss.

**Results:**

In total, 75 injuries were documented in 66 athletes during the Paralympic Games, with an injury incidence of 12.5 (95% CI 9.6–15.3) injuries per 1000 athlete days. The upper limb was the most injured anatomical region (55%), and athletes with spinal cord-related disorders incurred the most injuries (55%). At the Rio 2016 Paralympic Games, most injuries (88%) were associated with no time loss.

**Conclusion:**

This study's findings are consistent with existing literature describing the incidence observed in other wheelchair-based sports at the Paralympic Games. Injuries affecting the upper limb may indicate the influence of the nature of the sport and/or the additional load and inadequate rest of the upper limb in athletes who use wheelchairs outside of sporting activities, particularly in those with spinal cord-related disorders. These findings can assist medical staff in understanding the high incidence of low-burden upper limb injuries among athletes competing in wheelchair basketball.

In recent years, growth in Para sport participation has been observed, potentially because of increased awareness and accessibility to the sport. This growth has translated into increased athletes participating at the Paralympic Games, with 4378 athletes competing in the Rio 2016 Paralympic Games.^[1]^ This growth has also been observed in wheelchair basketball, representing the third largest sport for athletes at the Rio 2016 Paralympic Games.^[1]^ Despite the health benefits of sports participation, there may also be an increased risk of sustaining an injury, especially in individuals who have an underlying medical impairment.^[2,3]^

For wheelchair basketball, the rules are relatively similar to standard basketball, with some differences in dribbling and bouncing.^[4]^ The game is played on a basketball court with two teams of five players each. Players are classified between 1.0 and 4.5 points, depending on their physical ability (1.0 being the least physical ability). Each team is allowed to have 14 classification points in total on the court at one time.^[5]^ During a game, there are four quarters of ten minutes. Game points are scored by shooting the ball through the opponent’s hoop. Despite the similarities to standard basketball, the players are at risk of collisions with other players’ wheelchairs as well as different injury patterns related to wheelchair use.

Previous studies have described injuries in athletes participating in certain teams at tournaments or for a team over a season. In addition, one study investigated injuries during a tournament.^[4]^ The incidence of injuries reported in the literature varies, ranging from 12.0 and 12.8 injuries per 1000 athlete days seen at the London 2012 and Rio 2016 Paralympic games, respectively, to 68.9 injuries per 1000 athlete days at the 2018 Wheelchair Basketball World Championships (WBWC).^[1,4,6,7]^ The high incidence of injuries reported at the WBWC could be due to excellent compliance with reporting with a small medical team and the presence of one member of the study group at the venue every day.^[4]^ Wheelchair basketball was among the top five sports for the number of injuries sustained during the London 2012 (fifth highest) and Rio 2016 (third highest) Paralympic Games.^[1,6]^ These findings highlight the importance of monitoring injuries sustained in wheelchair basketball and addressing prevention of these injuries through informed injury prevention programs.^[4,7,8]^

This research is particularly important for Paralympic athletes since limited studies on the epidemiology of injuries are available among Paralympic athletes. In addition, the type of injuries, mechanisms, burden and onset can be unique depending upon athletes’ underlying impairment. It can also significantly impact their daily lives away from sports.^[5,6,9]^ The incidence of time-loss injuries at the WBWC was reportedly low at 5.5 (95% CI 1.7 to 9.3) injuries per 1000 athlete-days, and the high incidence of injuries could be due to the reporting of minor non-time loss injuries, including muscle spasms and skin lacerations.^[4]^ Nonetheless, this research can help to identify the patterns of injuries that occur and which athletes are at a higher risk based on their impairment types. Currently, no research describes the specific injuries sustained by Paralympic athletes in wheelchair basketball at the Paralympic Games.

This study analyses the incidence and characteristics of injuries sustained by athletes participating in wheelchair basketball at the London 2012 and Rio 2016 Paralympic Games, by sex, age, anatomical area, and impairment type. Based on these data, authorities involved in the game can develop informed injury prevention strategies for elite athletes and plan medical support structures for events.

## Methods

### Study design and setting

This study is a sub-study within the ongoing Paralympic injury and illness surveillance project, initiated at the London 2012 Paralympic Games.^[1]^ The larger study used the web-based injury and illness surveillance system (WEB-IISS) to document injuries.^[10,11]^ This sub-study looked at medical attention injuries sustained by athletes while participating in wheelchair basketball at the London 2012 and Rio 2016 Paralympic Games. The data includes all injuries that were recorded during the 14 days that covered the pre-competition and competition period, at both Paralympic Games. The larger ongoing study, as well as this sub-study, were approved by Stellenbosch University Health Research Ethics Committee (Reference numbers: N16/05/067 and S22/05/087, respectively).

### Data collection

The injury data were collected from three sources: 1) The International Paralympic Committee (IPC) master list, 2) Injury records from team medical staff via the WEB-IISS, and 3) Records from the Paralympic Village polyclinics and sports venues (except for Rio 2016 where polyclinic records were unavailable).^[1,10]^ Time loss was not recorded for injuries at the London 2012 Paralympic Games. The definition of an injury used was ‘any athlete incurring an injury requiring medical attention, regardless of the consequence with respect to absence from training or competition’.^[10]^ This is consistent with the International Olympic Committee (IOC) consensus statement and the Para translation of this consensus.^[12,13]^ If an athlete sustained multiple injuries, each injury was documented as a distinct injury encounter.

### Injury incidence, proportion and percentage

Team size was determined based on athletes on the IPC master list.^[1]^ These data were used to calculate the number of athlete days by multiplying the team size by the number of competition days.^[1,6]^ The injury incidence was calculated by dividing the number of injuries by the number of athlete days multiplied by 1000. The injury proportion (IP) of athletes that sustained an injury was calculated by dividing the number of athletes with an injury by the total number of athletes that participated in the sport multiplied by one hundred. Injury incidence and proportion were calculated for all injuries and categorised by sex, age group, anatomical area and onset pattern. Injury percentage was calculated by dividing the number of injuries by the overall number of injuries multiplied by 100. This was reported for impairment type and time loss.

The overall injury incidences and proportions for wheelchair basketball for each Paralympic Games have previously been published and are not novel to this study. Time loss was recorded for injuries, ranging from no days lost to the number of days an athlete missed training or competition.

### Statistical analysis

Descriptive statistical analyses were used to report demographic characteristics of the athletes in the form of counts and incidence, including total number of injuries, together with sex (male or female), age group (12–25, 26–34 and 35–75 years), anatomical region (upper limb, lower limb, head/face /neck, spine, chest/trunk/abdomen, unspecified) and pattern of onset (acute (sudden onset)/repetitive (sudden onset)/repetitive (gradual onset)). In addition, IPs were reported per impairment type and time loss from competition and/or training. Statistical analysis was performed using Microsoft Excel (Microsoft Excel 365, 2022, Redmond, Washington, USA).

## Results

### Participants

In total, 430 athletes participated in wheelchair basketball at both Paralympic Games. Table 1 describes the number of athletes by age group and sex who participated in wheelchair basketball at these Paralympic Games. Most of the athletes were male (58%), with the highest percentage falling into the 26–34 age group (47%).

### Overall injury incidence

Table 2 presents the total number of injuries, IP and incidence for these Paralympic Games, by age category and sex. Over the pre-competition period and competition period at both Games 75 injuries were recorded in 66 athletes (IP 15.3%). The overall incidence for wheelchair basketball was 12.5 (95% CI 9.6–15.3) injuries per 1000 athlete days.

### Incidence of injury by sex and age group

The incidence of injury by age category and sex is presented in Table 2. The highest incidence occurred in male athletes, although no direct comparisons were performed. In addition, the highest incidence was observed in athletes between the ages of 26 and 34 years.

### Injuries by onset

Table 3 describes the pattern of onset of injuries, showing the highest incidence was for acute (sudden onset) (7.8 (95% CI 5.6 – 10.0)). The IP for acute (sudden onset) injuries was 9.5% for the Paralympic Games.

### Incidence of injuries by anatomical area

The incidence of injuries sustained per anatomical area is presented in Table 4. Overall, injuries to the upper limb were the most frequent with an incidence of 6.8 (95% CI 4.7–8.9) per 1000 athlete days and an IP of 8.4%. The second highest IP was observed in the head/face/neck and lower limb areas at both Paralympic Games. The anatomical areas that had the highest incidences were the shoulder/arm/elbow complex (incidence of 3.5 (95% CI 2.0–5.0) per 1000 athlete days) followed by the wrist/hand/finger complex (incidence of 3.0 (95% CI 1.6–5.0) per 1000 athlete days). In the upper limb, most injuries were soft tissue lacerations, bruising and hematomas, and muscle and tendon injuries.

### Percentage of injuries by impairment type

Table 5 presents the injuries reported by athlete’s impairment types. Athletes with a spinal cord related disorders sustained the highest number of injuries (55%). The percentage of injuries found for athletes with neuromuscular disorders, limb deficiency or impaired passive range of motion, was similar across both Paralympic Games. More than half (n=22) the injuries to athletes with spinal cord related disorders were in the upper limb, of which 10 injuries involved the shoulder/arm/elbow, 12 involved the wrist/hand/finger and five involved the neck.

### Estimated time loss from injury

For the athletes that participated in the Rio 2016 Paralympic Games, the majority of injuries (36 of 41 injuries) resulted in no time loss (88%), three injuries resulted in one day of time loss to the injury (7%), while two injuries resulted in two and three days of time loss, respectively (5%).

## Discussion

To our knowledge, this study is the first to describe injuries requiring medical attention in wheelchair basketball over two Paralympic Games, highlighting four main findings: 1) injury incidence is similar to that in other wheelchair sports and higher than in non-wheelchair sports, 2) upper limb injuries are the most common, 3) spinal cord-related disorders are the most frequent impairment among injured athletes, and 4) most injuries did not result in time loss.

### Wheelchair basketball compared to other wheelchair sports

The overall injury incidence in wheelchair basketball (12.5 injuries per 1000 athlete days) aligns with other wheelchair sports, such as wheelchair tennis, rugby, and fencing, all of which feature high injury rates. This appears consistent with previous studies that investigated injuries in athletes with disabilities participating in wheelchair-based sports.^[1,4–7,14]^ All four of the mentioned wheelchair sports are consistently in the top 10 sports for injury incidence at the Paralympic Games, suggesting that the use of wheelchairs in sports is associated with a higher incidence of injuries compared to other Paralympic sports.^[1,6]^

The IP in wheelchair basketball is similar to wheelchair rugby and tennis but lower than in wheelchair fencing at both Games.^[1,6,10]^ Long-term studies show higher IP, indicating a potential for repetitive injuries, which warrants further longitudinal research.^[3]^

### Upper limb injuries are most common

The anatomical area with the highest incidence was the upper limb (incidence of 6.8). For upper limb injuries, most were reported in the shoulder/arm/elbow complex (incidence of 3.5) followed by the wrist/hand/finger complex (incidence of 3.0). Previous studies supported these findings of upper limb and shoulder injuries in wheelchair basketball.^[4,7,15,16]^ These findings were also consistent with findings in other wheelchair sports.^[8,17–19]^ Common diagnoses for the injuries in the shoulder were soft tissue, muscle and tendon injuries, including shoulder impingement and rotator cuff injuries. Injuries to the wrist/hand/ fingers also consisted of soft tissue, muscle and tendon injuries, joint injuries and lacerations/abrasions. Both these were consistent with previous research during the WBWC in 2018.^[4]^ The high incidence of upper limb injuries may be related to the nature of the sport, with a high burden on the upper limbs through repetitive strenuous overhead throwing and wheelchair propulsion, as well as upper limb contact sustained with other players and wheelchairs.^[8,16]^ It can also be associated with the type of impairments of athletes participating in wheelchair basketball, including lower limb and trunk impairment and reliance on the upper body in daily life and during sport.^[7,11]^ The daily reliance on shoulder movement for mobility for most individuals can make traditional treatment options (such as rest) difficult, making these injuries challenging to treat.^[7]^

### Injuries sustained by athletes with spinal cord related disorders

This study demonstrated that most injuries occurred in athletes with spinal cord-related disorders (55%). Previous research indicated that a significant portion of athletes involved in wheelchair basketball have spinal cord-related disorders (61%).^[2,3,5]^ When assessing the athlete population at the Rio 2016 Paralympic Games, athletes with limb deficiency experienced the highest injury rate at 30%, while spinal cord-related disorders accounted for only the third highest injury percentage at 20%, which is lower than in wheelchair basketball.^[1]^ The substantial number of athletes with spinal cord-related disorders participating in wheelchair basketball may help explain the increased injury incidence seen in this group compared to other Paralympic sports. Further investigation is necessary to outline the incidence by impairment to identify which impairments are most at risk for injury in wheelchair basketball.

### Minimal time loss reported

This study showed that most injuries in wheelchair basketball at the Rio 2016 Paralympic Games did not result in time lost from training and/or competition. This number exceeds the overall Paralympic time loss (75%).^[1]^ It may indicate that many of these injuries were minor or did not affect the athlete’s capacity to train or compete. This is consistent with previous research, indicating that injuries requiring time loss ranged from 6% to 33%.^[4,7,8,15]^ The high incidence of low-burden injuries may be part of the nature of the game with the use of wheelchairs. However, it is important to address minor injuries that do not result in time loss, as these injuries can limit an athlete’s performance and function in daily life.^[7,8]^ Furthermore, anticipated time loss was not available for the London 2012 Paralympic Games.

### Strengths and limitations

This study was the first to describe the injuries sustained by athletes participating in wheelchair basketball across multiple Paralympic Games. Limitations include a small sample size of injured athletes. Nevertheless, the findings are significant for beginning to understand the epidemiology of injuries in these athletes. Missing injury data for one athlete from the London 2012 Paralympic Games resulted in one unknown data point, and time-loss data from injuries during the London 2012 Paralympic Games were not collected. Data collection limitations and bias were beyond the scope of this sub-study. We hope this study will lay the foundation for larger, basketball-specific studies. The recording of time loss associated with injuries is recommended to make robust comparisons and allow insight into the severity and burden of injuries in this population.

## Conclusion

The incidence of injuries among athletes competing in wheelchair basketball at the London 2012 and Rio 2016 Paralympic Games was 12.5 (95% CI 9.6–15.3) injuries per 1,000 athlete days. This incidence was relatively high compared to other Paralympic sports while being similar to other wheelchair sports. Upper limb injuries were reported most frequently, and athletes with spinal cord-related disorders had the highest proportion of injuries in wheelchair basketball. Although injuries appear to have a high incidence, they result in a low burden of time loss. These data depict the injuries within this population of athletes and offer useful information for medical staff and organisations working with these athletes to guide areas for further research in injury prevention.

## Figures and Tables

**Table 1:** Demographic characteristics of athletes participating in wheelchair basketball at the London 2012 and Rio 2016 Paralympic Games

Age group (years)/sex	Total number of athletes (% of total number of athletes)

Male	250 (58%)
Female	180 (42%)

Age 12–25	93 (22%)
Age 26–34	202 (47%)
Age 35–75	135 (31%)

**All**	**430 (100%)**

**Table 2:** Combined Incidence of injury in wheelchair basketball at London 2012 and Rio 2016 Paralympic Games by sex and age

Games	Total no. Of injuries (% of total number of injuries)	No. Of athletes with an injury	Total no. Of athletes competing	Total number of athlete days	Injury proportion (%)	Injury incidence^*^

Male	49 (65%)	41	250	3500	16.4	14.0 (10.1–17.9)
Female	25 (34%)	24	180	2520	13.3	9.9 (6.0–13.8)
Unknown Sex	1 (1%)	1	-	-	-	-

Age 12–25	13 (18%)	12	93	1302	12.9	9.8 (4.5–15.2)
Age 26–34	38 (51%)	32	202	2828	15.8	13.4 (9.2–17.7)
Age 35–75	23 (31%)	25	135	1890	18.5	12.2 (7.2–17.1)
Unknown Age	1 (1%)	1	-	-	-	-

**Total**	**75 (100%)**	**66**	**430**	**6020**	**15.3**	**12.5 (9.6–15.3)**

**Table 3:** Incidence of injury in wheelchair basketball at London 2012 and Rio 2016 Paralympic Games by pattern of onset

Pattern of onset	Total injuries (% of total injuries)	Number of athletes with an injury	Injury proportion (%)	Injury incidence*

Acute (sudden onset)	47 (63%)	41	9.5	7.8 (5.6–10.0)
Repetitive (sudden onset)	11 (15%)	8	1.9	1.8 (0.7–2.9)
Repetitive (gradual onset)	16 (21%)	16	3.7	2.7 (1.4–4.0)
Unknown	1 (1%)	1	-	-

**Total**	**75 (100%)**	**66**	**15.3**	**12.5 (9.6–15.3)**

* number of injuries/1000 athlete days (95% CI)

**Table 4:** Incidence of injury in wheelchair basketball at London 2012 and Rio 2016 Paralympic Games by anatomical area

Anatomical area	Total (% of total injuries)	Number of athletes with an injury	Injury proportion (%)	Injury incidence*

**Upper Limb**	**41 (55%)**	36	**8.4**	**6.8 (4.7–8.9)**
Shoulder/arm/elbow	21 (28%)	19	4.4	3.5 (2.0–5.0)
Wrist/hand/finger	18 (24%)	17	4.0	3.0 (1.6–4.4)
Unspecified	2 (3%)	2	0.5	-

**Lower Limb**	**12 (16%)**	11	**2.6**	**2.0 (0.9–3.1)**
Ankle/foot/toe	6 (8%)	6	1.4	1.0 (0.2–1.8)
Knee	3 (4%)	3	0.7	-
Hip/groin/pelvis	2 (2.7%)	2	0.5	-
Unspecified	1 (1.3%)	1	0.2	-

**Head/Face/Neck**	**12 (16%)**	12	**2.8**	**2.0 (0.9–3.1)**
Neck	10 (13%)	10	2.3	1.7 (0.6–2.7)
Head/Face	2 (3%)	2	0.5	-

**Spines**	**6 (8%)**	6	**1.4**	**1.0 (0.2–1.8)**
Thoracic spine	3 (4%)	3	0.7	-
Lower back/Lumbar spine	3 (4%)	3	0.7	-

**Chest/Trunk/Abdomen**	**2 (3%)**	2	**0.5**	-
Abdomen/chest	1 (1%)	1	0.2	-
Unspecified	1 (1%)	1	0.2	-

**Unspecified**	2 (3%)	2	0.5	**-**

**Total**	**75 (100%)**	**66**	**15.3**	**12.5 (9.6–15.3)**

* number of injuries/1000 athlete days (95% CI)

**Table 5:** Percentage of injuries by impairment type for London 2012 and Rio 2016 Paralympic Games

Impairment	Total injuries	Number of athletes with an injury	Percentage of injuries by impairment type (%)

Spinal cord-related disorders	41	34	55
Neuromuscular disorders	11	10	15
Impaired passive range of motion	10	9	13
Limb deficiency	10	10	13
Unknown	3	3	4

**Total**	**75**	**66**	
